# Childhood Obesity — What We Can Learn From Existing Data on Societal Trends, Part 1

**Published:** 2004-12-15

**Authors:** Roland Sturm

**Affiliations:** RAND

## Abstract

The number of overweight and obese youth has increased in recent decades, and numerous theories on causes have been proposed. Yet almost no data are available to assess how the lives of children have changed during the “obesity epidemic.” What are children and adolescents now doing with their time that they did not do before? Are they participating less in sports? Watching more television? Doing more homework? Without tracking these broader societal changes, it is difficult to identify the most (and least) promising areas for interventions. This two-part report compiles trend data for several areas. Part 1 discusses trends in time use, homework, and media use; part 2 discusses trends in transportation, physical education, and diet.

The main findings of this article are the following: One, the free time of children has substantially declined because of increased time away from home, primarily in school, day care, and after-school programs. Two, participation in organized activities (including sports) has also increased. Three, unstructured playtime has decreased to make room for organized activities. Four, time spent in some sedentary activities like watching television, participating in conversations, or taking part in other passive leisure activities also declined just when obesity became a major concern. Five, increases in homework have not caused decreases in free time, contradicting a common belief in education circles.

## Introduction

The number of overweight and obese youth has been increasing dramatically in recent decades, and there is no sign that this trend is ending (1). Prevention may be one of the hallmarks of pediatric practice, but office-based counseling offers limited leverage to counter broader changes that affect the daily lives of children. Even though prevention and treatment in clinical settings have been the focus for interventions in the past, researchers now agree that trends in overweight arise from changes in social and environmental factors that need to be understood and modified for effective prevention (2,3). Many factors have been suggested as causes of the "obesity epidemic" among children — reduced physical education at school, increased homework loads, campus vending machines, television, larger portion sizes, fast-food restaurants, video games, and countless others. Yet virtually no data track how the lives of children have changed during the obesity epidemic; in fact, except for the National Health and Nutrition Examination Survey (NHANES), no reliable data exist to track weight increase among children (1).

This two-part report reviews data sources available for time trends, summarizes trend data that have been published (typically outside the health literature), and provides several new calculations. Finding comparable data across several years is difficult; finding such data across several decades is even more difficult. With purely cross-sectional data, we can only compare instantaneous snapshots against an ideal world, but what we really want to know is: What has changed since the time when childhood obesity was not a major public health problem? What are children and adolescents now doing with their time that they did not do before? How are they less physically active? Are they participating less in sports? Without tracking these broader societal changes, we will not easily identify the most promising areas for intervention.

When considering an array of information, it is often useful to start with a systematic review of the primary research literature. Systematic reviews are most useful when multiple studies pose similar research questions. Guidelines on clinical practice are often based on systematic reviews because many studies have identical research questions. An Institute of Medicine committee focused on preventing childhood obesity has taken the systematic review approach (4) and found more than 40,000 citations after searching the topics of obesity, overweight, body weight, dietary patterns, and physical activity. Unfortunately, this approach has not been very successful in identifying information about societal trends. Much of the primary research on societal trends does not focus on health, weight, or physical activity and contains no related key words in the abstracts. A seminal paper on changes in children's time use, for example, contains none of the words or word fragments "obesity," "overweight," "body weight," "diet," or "physical activity" anywhere in the text (5).

The approach taken here is, therefore, more eclectic by necessity. The research literature on childhood obesity has several themes, including transportation, media use, physical education, school hours, and diet. For this report, all data sets on children at the Inter-university Consortium for Political and Social Research — an organization of member institutions that archives social science data — were scanned for articles providing time-series data. Most sources were found, however, by the less systematic approach of identifying the main data sets and surveys used in the fields mentioned above and then determining whether those data could provide information on secular trends.

Analysis of time-use data is a first step toward understanding how economic incentives have altered behavior patterns that lead to weight gain. Indeed, time-use data for adults are so important in tracking societal trends that in 2003 the Bureau of Labor Statistics and the Census Bureau started to collect time-use data. Ideally, we would like to translate time use into energy expenditure and examine how this relationship contributes to weight gain, but the data are not detailed enough for this task. The next section of this study discusses existing time-use data.

When we try to understand the economic and technological forces behind changes in time use, few technological innovations have been more important in the lives of children and adolescents than the emergence and evolution of new communication technologies. Technologies like cable television, videos, computer games, and the World Wide Web have altered how children obtain information and entertainment, but how do they affect children's physical activity patterns or use of time? A later section of this article (Media) discusses available data, but unfortunately we can only calculate trend data for television watching. Only a single cross-sectional national survey (for 1999) provides a full assessment of all media use.

In education circles, the hypothesis that homework, or, more generally, the academic excellence movement, plays an important role in weight gain (and/or declining physical fitness) among children appears to be widely accepted, even outside the United States. In surveys conducted by the Chinese University of Hong Kong, the National University of Malaysia, Thailand's Mahidol University, and the Philippine Food and Nutrition Research Centre, by far the most common reason students gave for not participating more in physical activity was homework, cited 1.5 times more often than the runner-up, heat/weather (6). The final section of this article reviews data sources to investigate the hypothesis that an increase in homework has contributed to weight gain and/or lack of physical activity.

The second part of this report in Volume 2, Issue 2 of *Preventing Chronic Disease* will look at data for three other areas: transportation, physical education, and diet.

## Time Use

We first review existing data on how American children spend their time. There are several ways of assessing time use, but numerous methodological studies show that the best way to collect large-scale time-use data is to use time-diary data, where individuals describe what they have done during the past day (7). Relatively comparable time-use data for adults have been collected approximately every decade since the 1960s, enabling researchers to paint a broad picture of how adult lives and physical activity have changed in the past 40 years (8,9). Time-use data for children are sparse, but researchers from the University of Michigan fielded two surveys in 1981 and 1997 (5,10). Data presented in this article are based on calculations made from data provided in detailed tables published in 2001 (5).

Both University of Michigan surveys used 24-hour time diaries with similar methodology. The 1981 data are based on time diaries of 222 children aged two to 12 years. Each child provided data for one school day and one nonschool day. The data are nationally representative (weighted for sampling probability and post-stratification factor) and have been used in a variety of other reports, primarily in education. The 1997 survey was an addition to the Panel Study of Income Dynamics, a representative sample of U.S. men, women, children, and their families. The study consisted of interviews of 2380 households containing 3563 youth and had a response rate of 88%. Post-stratification weights based on the 1997 Current Population Survey are used to make the data nationally representative, and sampling weights adjust for survey design. Subsetting to children aged three to 12 years with complete data resulted in 2119 observations.

### Free/discretionary time has declined

**Figure 1:**
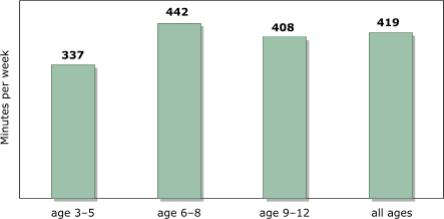
Decline in discretionary time (in minutes per week) between 1981 and 1997 among U.S. children aged three to 12 years. Calculations based on data from Hofferth and Sandberg (5).

The free time of children as a proportion of the total weekly time of 168 hours declined by approximately 12% from 1981 to 1997, if time spent eating, sleeping, in personal care (e.g., preparing to go places, packing, getting dressed), in school, and in child care is subtracted from the total. The decline in free time — between seven and eight hours per week, depending on how we treat study time or household work — is largely due to increased time spent in school and child care and to a lesser extent to increased personal-care time. Figure 1 shows that this decline occurred across all age groups; data are significant at *P* < .01.

**Table:** A text description of this chart is also available

**Figure 2:**
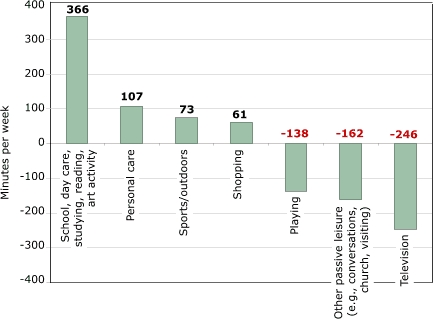
Changes in time (in minutes per week) spent on activities between 1981 to 1997 by U.S. children aged three to 12 years. Calculations based on data from Hofferth and Sandberg (5).

Time spent in school increased about two hours per week, or 25 minutes per school day, from 24 hours and 45 minutes to 26 hours and 48 minutes in an average week. Day-care time increased from about 14 minutes to almost three hours per week because both a larger share of children used day care and children in day care spent more time in it. In Figure 2, we add other academic activities that time-use researchers often consider discretionary but that have similar purposes (and physical activity levels): primarily reading and studying at home. Personal-care time also increased over the same period, from more than six hours per week to eight hours per week. Time-use researchers have hypothesized that as children spend more time away from home, they need more time to get ready to do so (5). Smaller changes occurred in time spent eating, which decreased, and sleeping/napping, which increased by the same amount (not shown). The decline in eating time parallels a decline in the frequency with which families sit down together to share a family meal (11). More important changes occurred in the composition of children's free time. While time spent in some categories decreased by more than the average 12%, time spent in other categories increased. Time spent viewing television as a primary activity declined by the greatest percentage — by 23%, or about four hours per week (Figure 2, *P* < .001). This decline did not take place because of a decrease in the proportion who watched television (because almost all children watched television during both 1981 and 1997); instead, the decline took place because of a reduction of television time among watchers.

Several other sedentary activities declined significantly and proportionately more than discretionary time overall: church attendance, youth-group participation, passive leisure, and other household conversations. In Figure 2, this group of activities represents the second largest group of declines. But two sedentary activities at home, reading and studying (grouped with school/day care in Figure 2), also increased.

Time spent in hobbies, organized sports, and arts activities increased, reflecting an overall trend toward structured activities and a decline in unstructured activities. Figure 2 groups only the more active categories together (sports/outdoors), and they show a substantial increase. Sports increased significantly for children younger than nine years; other categories did not change significantly. Hobbies/arts, which include dance and music lessons (not shown here), are more active than watching television or other passive leisure and are perhaps more comparable to playtime.

**Table:** A text description of this chart is also available

Hofferth and Sandberg analyzed the role of shifting demographics and maternal employment and found that few of the changes in time use could be linked to either factor (5). While some time use differed according to maternal education, family size, and family composition in 1981, different socioeconomic groups tended to become more similar rather than more different in time use. Thus, the increase in time spent in school (which includes after-school care) reflects changing social preferences for greater use of schools and school activities.

### Age differences

**Figure 3:**
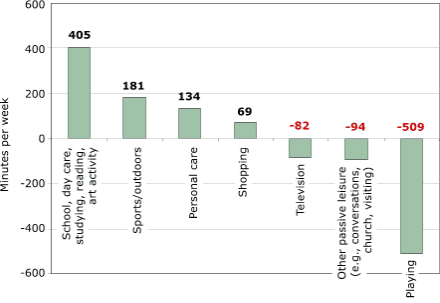
Changes in time (in minutes per week) spent on activities between 1981 and 1997 by U.S. children aged three to five years. Calculations based on data from Hofferth and Sandberg (5).

**Figure 4:**
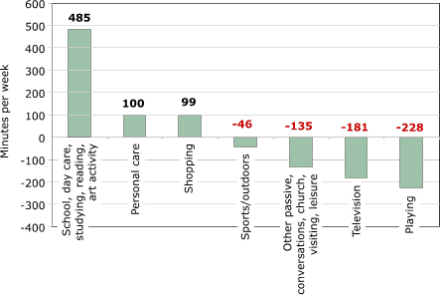
Changes in time (in minutes per week) spent between 1981 and 1997 on activities by U.S. children aged six to eight years. Calculations based on data from Hofferth and Sandberg (5).

**Figure 5:**
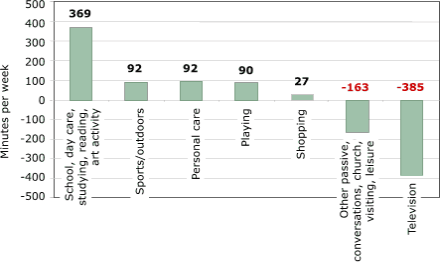
Changes in time (in minutes per week) spent between 1981 to 1997 on activities by U.S. children aged nine to 12 years. Calculations based on data from Hofferth and Sandberg (5).

Some notable differences in time use across different age groups suggest different levers for interventions by age group. For children aged three to five years (Figure 3) and children aged six to eight (Figure 4), the largest decline in time use is in playtime; for children aged nine to 12 years, the largest decline is in television watching (Figure 5). While playtime declined overall, it actually increased among children aged nine to 12. This increase may reflect more video- and computer-game use among this age group. The largest decline for children aged nine to 12 was in television watching, but household conversations and other passive leisure also declined more in this group than in other age groups. However, these declines may be countered by increases in sedentary playtime; time spent in this category grew by 1.5 hours per week in this age group (Figure 5). The declines and increases may represent trade-offs between different forms of media use, but time diaries do not reveal this level of detail.

**Table:** A text description of this chart is also available

While the dominant change is in the increase of time spent in school or day care away from home, time spent studying at home increased significantly among children aged six to eight, and time spent reading increased significantly among children aged three to five. The proportion of children aged three to five who spent time reading or being read to doubled between 1981 and 1997. Increased reading among this age group probably reflects increasing parental concern about preparing children for school. Increased enrollment in day-care centers and preschools may also be associated with children reading at early ages. We will examine homework loads more closely in a later section to investigate claims in the popular press and the education field that the homework burden has increased by so much that it now constitutes an enormous time burden on students and families, preventing them from engaging in other activities. The time-use data from the Michigan group is often used as a key piece in this argument, so it is worth keeping the magnitudes in mind: 76 minutes of the 485 additional minutes per week spent in school or other learning activities among children aged six to eight (Figure 4) were designated toward homework. Among children aged nine to 12, however, the increase in homework was not statistically significant, and the point estimate was an increase of 19 minutes for studying at home out of the 369 additional minutes per week in learning activities (Figure 5).

Figure 4 shows that sports/outdoor time did not increase significantly among children aged six to eight, representing a different pattern than other age groups. The major reason for the significant increase among children aged three to five was the increased proportion engaging in sports, which almost doubled over the period.

**Table:** A text description of this chart is also available

**Table:** A text description of this chart is also available

In summary, the largest change in children's time use in the past two decades was a decline in discretionary/free time, paralleled by an increase in school or day care and personal care. Particularly noteworthy are significant declines in many categories of passive leisure (television, conversations, other passive leisure) and increases (statistically insignificant) in sports/outdoor time. Increases in sports participation were largest among children of non-working mothers; thus, these increases did not result from increases in maternal employment (5,10). Children with less time to play mostly reflect decreased time spent at home. Children may be playing in their preschool programs, and they may have some free time at school, so the level of aggregation presented here provides only a partial picture of children's time and activity levels. Sports/outdoor time outside school is increasing, mainly among preschool children, but the increase in sports is significant for both the three-to-five and nine-to-12 age groups. Given the large increase in time children spend in school or day care, it is also important that children have enough physical activity in those settings. Moreover, the amount of time children participate in more physically challenging activities in school or day-care settings should have increased over time, corresponding to the total increase that children are now away from home. To what extent this has or has not occurred requires different data.

## Media

The first complete national data on media use among American youth were collected in 1999 by the Kaiser Family Foundation project, Kids & Media @ The New Millennium (12). Other surveys have examined children's use of selected media (most commonly television watching), but no data allow us to track media use comprehensively over time.

Despite interest in new media (e.g., computers, video games), television remains by far the dominant medium (Table). The impact of computers and video games on sedentary behavior is probably not very large, especially when compared with television, as they together comprise only about 10% of the average daily media budget of children aged two to 18. There are, however, large differences by age and sex. Children younger than eight years spent a negligible amount of time on video games or computers in 1998, but boys aged eight to 13 averaged 47 minutes per day playing video games (13). On the other hand, children aged eight to 12 also experienced the largest decline (approximately 50 minutes per day) in television watching between 1981 and 1997.

No comparable surveys track the latest changes for all children, although it is likely that computer use has increased since the Kaiser Family Foundation survey. Video gaming may or may not have peaked already by 1999. A new survey fielded in 2003 for children younger than seven years found that one in five children aged four to six plays computer games in a typical day (14). A new trend certainly is that some computers and video games target preschoolers.

Television has been around for a much longer time and because of its continuing dominance, it has received more attention. Television may contribute directly to obesity by reducing energy expenditure through displacing physical activity or indirectly by increasing dietary intake — through snacking during viewing or changing eating patterns caused by food advertising. Numerous cross-sectional studies found significant positive associations between television viewing and youth obesity; prospective studies include some null findings, but a randomized trial confirmed that a reduction in television watching can reduce weight gain (15-17).

**Figure 6:**
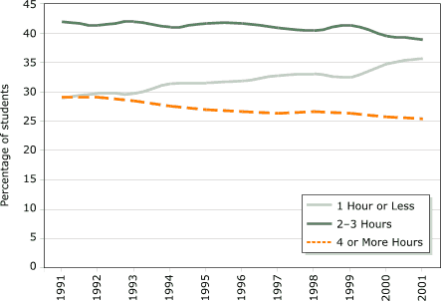
Percentage of teenagers who spend one hour or less, two to three hours, or four hours or more watching television on average weekday, 1991–2001. Analysis based on annual data from Monitoring the Future (18). Reprinted with permission from Child Trends.

Assessing time trends, even for television, is difficult because small differences in methods across different surveys create methods effects that far exceed real underlying changes. Nevertheless, we can compile some consistent time trends. The time-diary data suggest that children under 12 are now watching less television than they did in the past, a decline of about 23%, or about six hours per week between 1981 and 1997 (Figure 2). The Monitoring the Future survey confirms that this trend also holds for adolescents (Figure 6): there has been a substantial decline in heavy television watching and an increase in the proportion of adolescents watching one hour or less daily (18). Monitoring the Future is a large and nationally representative study; the documented decline is therefore very likely to represent a true effect. There are no conclusive data on whether the large decline in television viewing is more than offset or only partially offset by new-media use (e.g., video games, computer).

**Table:** A text description of this chart is also available

## Studying at Home

Many news reports and academic and popular books claim that homework overburdens children and limits learning with lack of physical activity and weight gain as major secondary consequences (19). Although most of the evidence for this idea is anecdotal, we noted above that time spent on home study by children aged six to eight did increase between 1981 and 1997. This fact has been cited in a number of news reports and in the book *The End of Homework*, which is subtitled, *How Homework Disrupts Families, Overburdens Children, and Limits Learning* (19).

Gill and Schlossman used several national surveys to provide a 50-year perspective on time spent on homework; a summary of their main findings follows (20).

**Figure 7:**
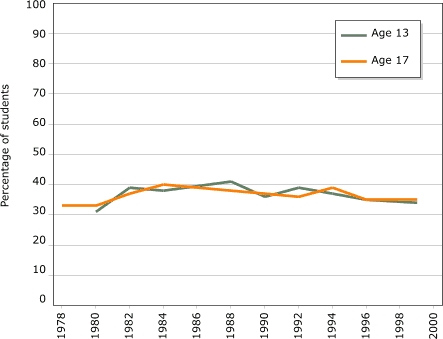
Proportion of U.S. students doing one hour or more of homework. Data available for every other year starting 1978–1996 (except 1986), plus 1999; data not available for children aged 13 in 1978. Data from Gill and Schlossman (20). Copyright 2003 by the American Educational Research Association. Reprinted with permission from publisher.

The most systematic evidence on homework time at multiple grade levels across the country is found in answers to background questions asked of students taking the National Assessment of Educational Progress (NAEP). The data are nationally representative and based on large samples. Most variables show little change. Variables that do change, however, tend to show increases in the 1970s and early 1980s and declines in the 1990s (20). Figure 7 shows the trends: increases in the early 1980s were followed by a decline in the 1990s, resulting in figures for 1999 that are similar to those for 1980. Overall, increased time spent studying at home does not appear to be related to the obesity epidemic among youth.

**Table:** A text description of this chart is also available

**Figure 8:**
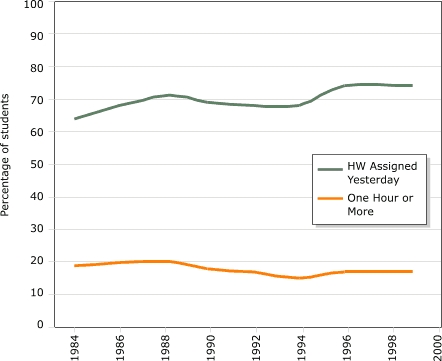
Homework trends for children aged nine years. Data available every other year 1984–1996 (except 1986), plus 1999. Data from Gill and Schlossman (20). Copyright 2003 by the American Educational Research Association. Reprinted with permission from publisher.

Homework loads for elementary-level students deserve further analysis, however. The NAEP data collection for children aged nine (third or fourth grade) started only in 1984. Figure 8 shows two indicators: rates of homework assignment and the proportion of students doing more than one hour of homework the night before the survey. Clearly, the number of students with homework assigned the prior day has increased, consistent with an increase in average study time, but the one-hour-or-more trend line — flat or even declining — indicates that the daily time increase cannot have been very large. Regular daily assignments may also distribute work more evenly throughout the week and therefore decrease the probability of working one hour or more on any given day.

**Table:** A text description of this chart is also available

Combining several different data sets, we can track the proportion of high school students doing substantial homework over the half-century from 1948 though 1999. High school students during the late 1940s and early 1950s studied no more or less than their counterparts did in the 1970s, 1980s, and 1990s; only during the 1960s did homework time temporarily increase (20).

## Summary

In contrast to adults, who now have more free time than in the past, children have less free time than previously because of increased time away from home, primarily in school, day care, and after-school programs (5,8,9). Participation in organized activities (including sports) also increased. Time spent in many sedentary activities — television viewing, conversations, or other passive leisure — declined just when obesity became a major concern. Unstructured playtime also declined except for in older children, but it is not clear whether this playtime was sedentary or active. The role of new media is not fully clear, although it is unlikely to have played a substantial role prior to 1999, except for children eight to 12 who spent a significant amount of time playing video games (considered unstructured playtime in the time-use data). But this age group also watched much less television at the turn of the century than in the 1980s.

Increased homework burdens and time studying at home have not caused a decrease in free time, contradicting a common belief in education circles. The great majority of American children at all grade levels now spend less than one hour studying on a typical day — an amount that has not changed substantially for at least 20 years. Compared to the large changes in other uses of time, it appears unlikely that changes in homework have altered the activity levels of children.

As time in structured settings away from home increases, so does the importance of physical activity in those settings. A substantial percentage of youth (about one third of high school students) is insufficiently active. An increase in structured time offers opportunities for interventions that may be more successful at expanding the number of youth who meet minimum-guideline criteria for strenuous physical activity than interventions targeted at diverse and unstructured home environments. In Part 2 of this report, we will look at trends in transportation, physical education, and diet.

## Figures and Tables

**Table 1:** Media Use Among U.S. Youth (Hours per Day), 1999*

** **	**2-18 year-olds**			**2-7 year-olds**			**8-18 year-olds**		

**Medium**	**White**	**Black**	**Hispanic**	**White**	**Black**	**Hispanic**	**White**	**Black**	**Hispanic**
**Total media exposure**	6:00^a^	7:56^b^	7:05^c^	4:04^a^	4:59^b^	4:25^a^	7:16^a^	9:52^b^	9:02^b^
**Television**	2:22^a^	3:56^b^	3:31^c^	1:43^a^	2:46^b^	2:20^b^	2:47^a^	4:41^b^	3:50^c^
**Taped television shows**	0:09^a^	0:17^b^	0:11^a^	0:04	0:02	0:02	0:12^a^	0:27^b^	0:18^c^
**Videotapes**	0:28	0:30	0:29	0:28	0:27	0:22	0:28	0:32	0:34
**Movies**	0:08^a^	0:19^b^	0:21^b^	0:01^a^	0:04^b^	0:01^ab^	0:13^a^	0:29^b^	0:35^b^
**Video games**	0:17^a^	0:25^b^	0:24^b^	0:08	0:08	0:09	0:23^a^	0:35^b^	0:35^b^
**Print media**	0:45^a^	0:45^a^	0:37^b^	0:47a^a^	0:42a^b^	0:38^b^	0:43^a^	0:47^a^	0:35^b^
**Radio**	0:38	0:40	0:43	0:22^a^	0:32^b^	0:25^ab^	0:49	0:45	0:56
**CDs and tapes**	0:50	0:43	0:49	0:22^a^	0:13^b^	0:23^ab^	1:09	1:03	1:08
**Computer**	0:22	0:20	0:19	0:07^a^	0:04^b^	0:04^ab^	0:31	0:31	0:29

* Within each row and age subgroup, only those mean times that do not share a common superscript differ from one another with statistical reliability. Those mean times without a superscript, or those that share a common superscript, do not differ by a large enough margin to ensure statistical reliability. Total media exposure is the sum of the amount of time children spend with each type of media. Data from Table 8c in Kids & Media @ The New Millennium (12) reprinted with permission from the Henry J. Kaiser Family Foundation.
