# Errata, Vol. 2: No. 1

**Published:** 2005-03-15

In the article "Childhood Obesity — What We Can Learn From Existing Data on Societal Trends, Part 1," some of the categories along the x-axis in Figures 4 and 5 were incorrectly labeled. In Figure 4, the categories "Sports/Outdoors," "Personal care," "Shopping," "Television," and "Other passive leisure (e.g., conversations, church, visiting)" were incorrectly labeled. In Figure 5, the categories "Shopping," "Television," and "Playing" were incorrectly labeled. The figures were corrected on our Web site on December 20, 2004 and appear online at http://www.cdc.gov/pcd/issues/2005/jan/04_0038.htm. We regret any confusion these errors may have caused.

In addition, the article "A Catalog of Biases in Questionnaires" contained an error in the example of a recommended question that prevents bias from overlapping intervals. The recommended question and response categories should be:

*How many cigarettes do you smoke per day?*[    ] None    [    ] 1–4    [    ] 5–24    [    ] 25 or more

The response category "1–4" appeared in the article as "4 or less." The error was corrected on our Web site on January 21, 2005 and appears online at http://www.cdc.gov/pcd/issues/2005/jan/04_0050.htm. We regret any confusion this error may have caused.

